# Highly conductive, conformable ionic laser-induced graphene electrodes for flexible iontronic devices

**DOI:** 10.1038/s41598-024-55082-w

**Published:** 2024-02-26

**Authors:** So Young Kim, Ji Hong Kim, Kyeong Nam Kim, Hayoung Oh, Sung Myung, Do Hwan Kim

**Affiliations:** 1 Department of Chemical Engineering, Hanyang University, Seoul, 04763 Republic of Korea; 2 Division of Energy Technology, DGIST, Daegu, 42988 Republic of Korea; 3 Thin Film Material Research Center, Korea Research Institute of Chemical Technology (KRICT), Daejeon, 34114 Republic of Korea; 4 Institute of Nano Science and Technology, Hanyang University, Seoul, 04763 Republic of Korea; 5 Clean-Energy Research Institute, Hanyang University, Seoul, 04763 Republic of Korea

**Keywords:** Mechanical and structural properties and devices, Electronic properties and devices

## Abstract

Iontronic devices, recognized for user-friendly soft electronics, establish an electrical double layer (EDL) at the interface between ion gels and electrodes, significantly influencing device performance. Despite extensive research on ion gels and diverse electrode materials, achieving a stable interfacial formation remains a persistent challenge. In this work, we report a solution to address this challenge by employing CO_2_ irradiation as a bottom-up methodology to directly fabricate highly conductive, conformable laser-induced graphene (LIG) electrodes on a polyimide (PI)-based ion gel. The PI ion gel exhibits exceptional EDL formation at the electrode interface, primarily attributable to efficient ion migration. Particularly, ionic laser-induced graphene (i-LIG) electrodes, derived from the PI ion gel as a precursor, yield high-quality graphene with enhanced crystallinity and an expanded porous structure in the upward direction. This outcome is achieved through a pronounced thermal transfer effect and intercalation phenomenon between graphene layers, facilitated by the presence of ionic liquids (ILs) within the PI ion gel. Ultimately, in comparison to alternative soft electrode-based vertical capacitors, the utilization of i-LIGs and PI ion gels in the vertical capacitor demonstrates reduced interfacial resistance and increased EDL capacitance, emphasizing the extensive potential of iontronic devices. These results not only highlight these features but also introduce a new perspective for advancing next-generation iontronic devices.

## Introduction

With the advancement of user-friendly soft electronics, iontronic devices have garnered significant attention across diverse domains, including applications such as wearable sensors^1^, energy storage devices^2^, bioelectronics^3^, actuators^4^, and transistor-based integrated circuits^5^. Notably, flexible iontronic devices feature ionic gel as their primary constituent, offering a unique combination of low modulus and low operational voltage that can be achieved by efficiently manipulating ion mobility within the ionic gel matrix. In particular, iontronic devices can achieve efficient operation even at low voltages due to their ability to establish an electric double layer (EDL) at the interface between the ionic gels and the electrodes, a characteristic inherent to ionic gel-based iontronic devices. This EDL effectively acts as a nanometer-thick capacitor, boasting a capacitance exceeding 1 μF cm^−2^, indicative of superior capacitive performance^6^. Unlike conventional dielectrics, the formation of the EDL at the ionic gel-electrode contact interface can be achieved using low voltages (< 3 V), rendering it an ideal choice for the development of energy-efficient, user-friendly, low-power flexible devices^7^. The crucial factor determining device performance is the electrode's ability to establish the EDL at the ionic gel interface, enabling the creation of high-performance iontronic devices.

As a result, extensive research has been conducted on flexible electrodes to enhance the performance of iontronic devices^8^. Previous studies have explored various electrode materials^9–11^, including Poly(3,4-ethylenedioxythiophene):polystyrene sulfonate (PEDOT:PSS), carbon nanotubes (CNTs), graphene, and silver nanowires(AgNWs), as well as the incorporation of additives^12^ and binders^13^. Additionally, researchers have focused on controlling synthesis and processing methods to ensure optimal electrical and mechanical properties, as well as stable interfaces^14^. While considering carbon-based electrodes, graphene has emerged as a standout choice due to its exceptional electrical conductivity and mechanical properties. Nonetheless, integrating graphene into the electrode layer of a device necessitates a complex, multi-step process involving intricate synthesis techniques^15^, inking^16^, and transfer processes^17^ for electrode formation. Furthermore, employing photolithography to create patterned electrodes introduces additional challenges when applied to various flexible devices. Additionally, when utilizing a solution-based process, limitations arise due to the degradation of the graphene electrode's electrical characteristics, resulting from the inclusion of binders or surfactants. This is because these additives are essential for enhancing the printability and stability of graphene inks.

Recently, laser-induced graphene (LIG) has gained considerable attention as a promising alternative to address the limitations associated with conventional carbon electrode materials. Consequently, various studies have explored ways to advance flexible iontronic device by harnessing dynamic behavior occurring at the interface between LIG and ionic gel. However, mechanical stability remains a concern due to the formation process involving drop casting^18^, spin coating^19^, and transfer^20^ of ion gels onto delicate LIG electrodes. Therefore, to establish a stable EDL at the interface, there is a need to explore a bottom-up direct method for electrode formation on the ionic gel, a method that has not yet been reported. In this regard, novel methodology to form the graphene electrode on top of the ion gel should be investigated thoroughly.

Here, we present a novel approach to directly synthesize LIG electrode on polyimide (PI)-ion gels using CO_2_ laser irradiation. The PI-ion gel we propose is formulated with PI and ionic liquids (ILs). Specifically it contains the cation–anion pair 1-ethyl-3-methylimidazolium bis(trifluoromethyl-sulfonyl)imide ([EMIM]^+^[TFSI]^−^). By modulating the IL concentration within this ion gel, we have effectively improved its ion transport properties. Furthermore, ionic laser-induced graphene (i-LIG) electrodes derived from the PI ion gel incorporating the IL exhibits an extended three-dimensional porous structure in an upward direction, presenting exceptional graphene qualities, including minimal defects and improved crystallinity. Finally, the capacitors utilizing i-LIGs and PI ion gels exhibit stable interfacial formation compared to traditional flexible electrode capacitors, showing a relatively high EDL capacitance based on low contact resistance and a large surface area. We anticipate that these results will offer an innovative and novel approach to advancing future iontronic devices driven by EDL capacitance.

## Results and discussion

**Figure 1:**
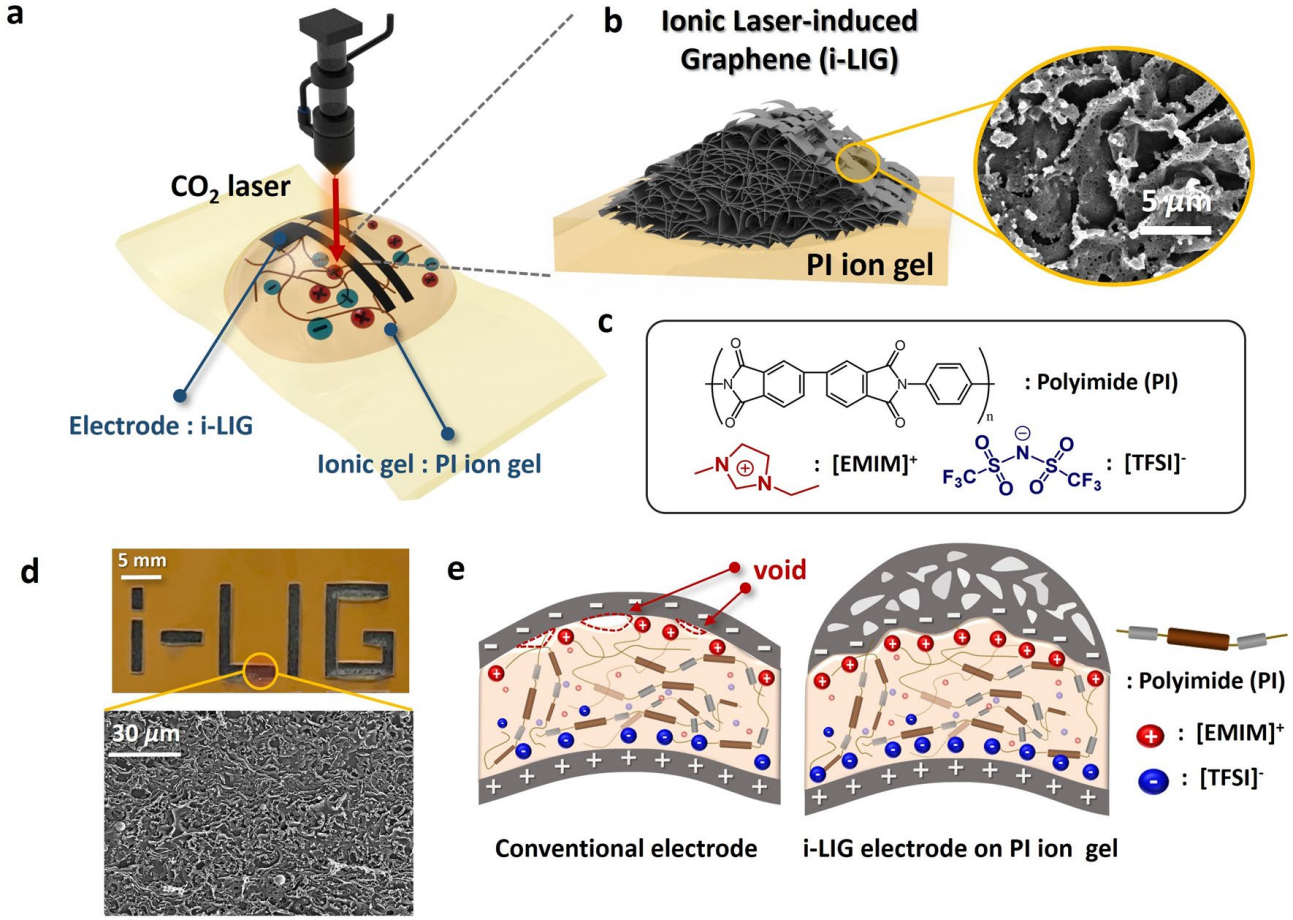
(**a**) Schematic illustration of the one-step CO_2_ laser irradiation process to convert the PI ion gel into i-LIG. (**b**) Schematic representation of i-LIG onto PI ion gel. (inset: FE-SEM image of i-LIG) (**c**) Chemical molecular structures of PI ion gel, comprising polyimide, IL ([EMIM]^+^[TFSI]^-^). (**d**) Photograph of i-LIG pattern on PI ion gel (top), and a FE-SEM image of i-LIG (bottom). (**e**) Illustration comparing the ion gel interface with i-LIG to a conventional electrode.

Figure 1a visually illustrates the conversion of our designed PI ion gel into i-LIG through CO_2_ laser irradiation. The cross-sectional morphology of the resulting i-LIG, as shown in Fig. 1b, reveals a porous three-dimensional structure formed through photothermal and rearrangement processes of the ion gel under CO_2_ laser irradiation. The corresponding FE-SEM image shows the three-dimensional porous morphology of the resulting i-LIG electrode. Notably, the morphology of i-LIG may exhibit variations based on distinct laser irradiation conditions and precursor materials^21^. Figure 1c depicts the molecular structures constituting the i-LIG precursor, derived from the PI ion gel, where PI acts as the polymer matrix and IL ([EMIM]^+^[TFSI]^−^) serves as the ionic material. The ion gel film is created by incorporating ILs into the PI precursor solution, followed by continuous heat treatment to form the final film. When CO_2_ laser irradiation is applied to the ion gel film, as illustrated in the upper section of Fig. 1d, various electrode patterns, including alphabets, can be formed. The resultant LIG electrode exhibits a porous three-dimensional morphology, as shown in the lower part of the same figure. Our proposed PI ion gel and i-LIG electrode interface, as depicted in Fig. 1e, can demonstrate superior interfacial characteristics compared to conventional electrodes and the ion gel interface. In contrast to conventional electrodes (left side of Fig. 1e), the i-LIG electrode, directly synthesized on the ion gel (right side of Fig. 1e), forms a flexible electrode layer without voids or defects. This results from the direct conversion of the PI ion gel into the i-LIG electrode through CO_2_ laser irradiation. Consequently, our proposed PI ion gel and the resulting i-LIG electrode can achieve a stable interfacial formation compared to other electrode and ion gel interfaces, ensuring enhanced ion transport characteristics and a higher EDL capacitance.

**Figure 2:**
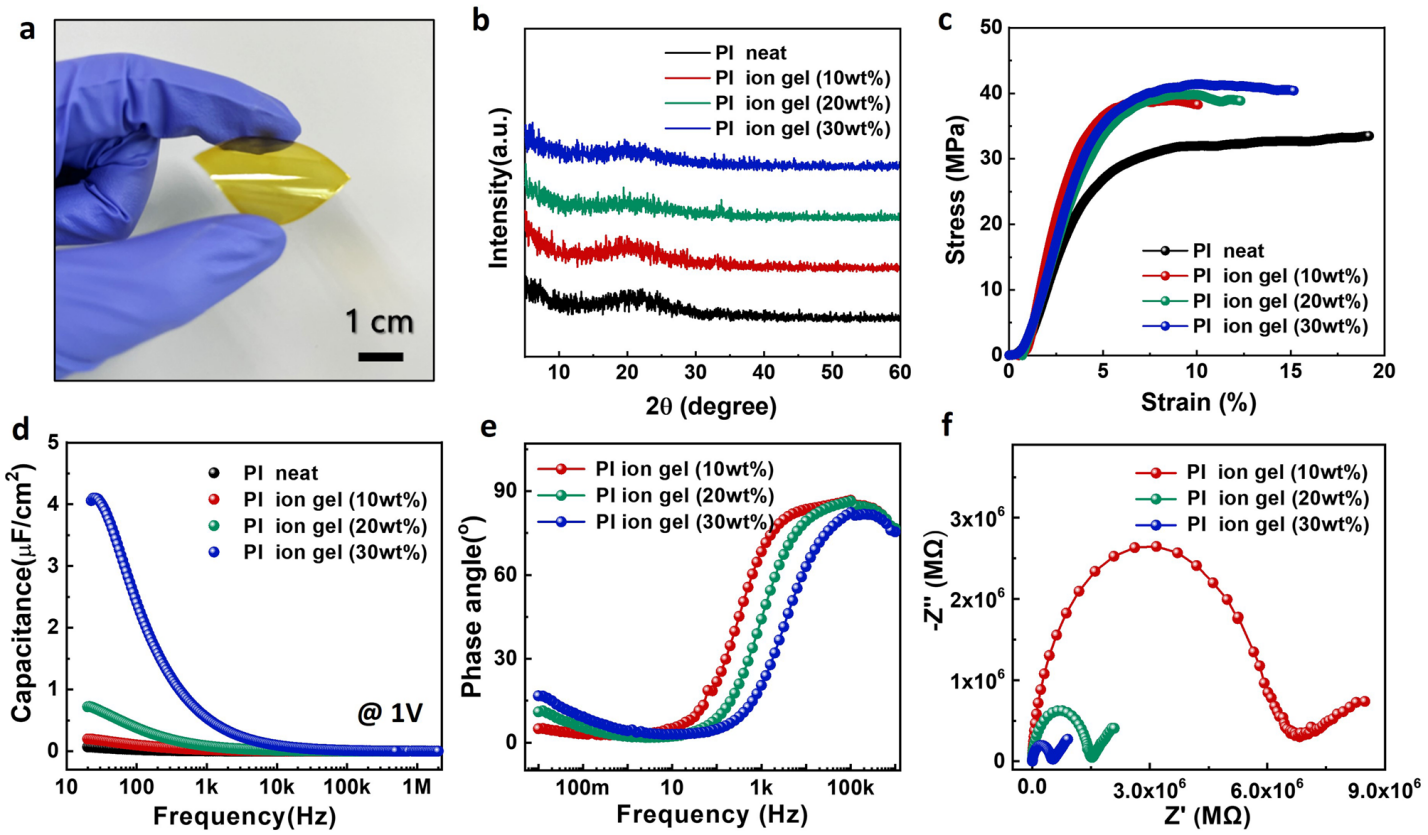
Mechanical, structural and electrochemical properties of PI ion gel (**a**) Photograph of free-standing PI ion gel film. (**b**) XRD patterns of PI neat and PI ion gel as function of ILs concentrations (10–30 wt%). (**c**) Stress–strain curves of PI neat, PI ion gel as function of ILs concentrations (10–30 wt%). (**d**) Capacitance-frequency of PI ion as function of ILs concentrations (10–30 wt%). (**e**) Phase angle-frequency of PI ion gel as function of ILs concentrations (10–30 wt%). (**f**) Impedance Nyquist plots (imaginary part − Z′′ as a function of real part Z′) of PI ion gel as function of ILs concentrations (10–30 wt%).

We initially investigated the distinctive mechanical, structural, and electrochemical properties of PI ion gel films (Fig. 2). Figure 2a presents standalone PI ion gel films, distinguished by their flexibility and a noticeable yellow tint. The thickness of these ion gel films is approximately 800 µm, and it remains consistent across all ion concentrations. To probe the internal crystal structure of these films, X-ray diffraction (XRD) measurements were conducted. As depicted in the XRD spectra in Fig. 2b, ion gel films consistently exhibit a prominent, broad peak at 21°, regardless of the IL concentration (0–30 wt%). This suggests the incorporation of ILs into the amorphous segments of the PI polymer matrix, with no observable changes to the crystalline structure. We then examined the mechanical properties through Stress–Strain (S–S) curves (Fig. 2c and Figure S1). The Young's modulus of PI ion gels shows minimal variation with IL concentration, while elongation tends to decrease compared to PI neat. Unlike conventional ion gels, ILs within PI ion gels primarily exist in amorphous segments, causing minimal changes to the Young's modulus, with some acting as cross-linking agents and leading to a slight decrease in elongation^22^. Furthermore, to evaluate ion migration and EDL formation within PI ion gels, we conducted comprehensive electrical and electrochemical analyses. Similar to conventional ion gels, the capacitance of PI ion gels increases with IL concentration inside the gels (Fig. 2d and Figure S2). Notably, the ion gel (30 wt%) demonstrates high capacitance, reaching up to 4 μF/cm^2^ at 20 Hz. This implies sufficient time for effective ion migration and EDL formation at lower frequencies. Additionally, the increased phase angle in the low-frequency range for PI ion gels indicates enhanced capacitor characteristics due to effective ion movement (Fig. 2e). Finally, Fig. 2f presents an impedance spectroscopy (EIS) Nyquist plot of PI ion gels, revealing a decrease in impedance and an increase in ionic conductivity with a rise in IL concentration. These findings provide evidence that the enhanced ion concentration within ion gels is directly associated with improved ionic conductivity.

**Figure 3:**
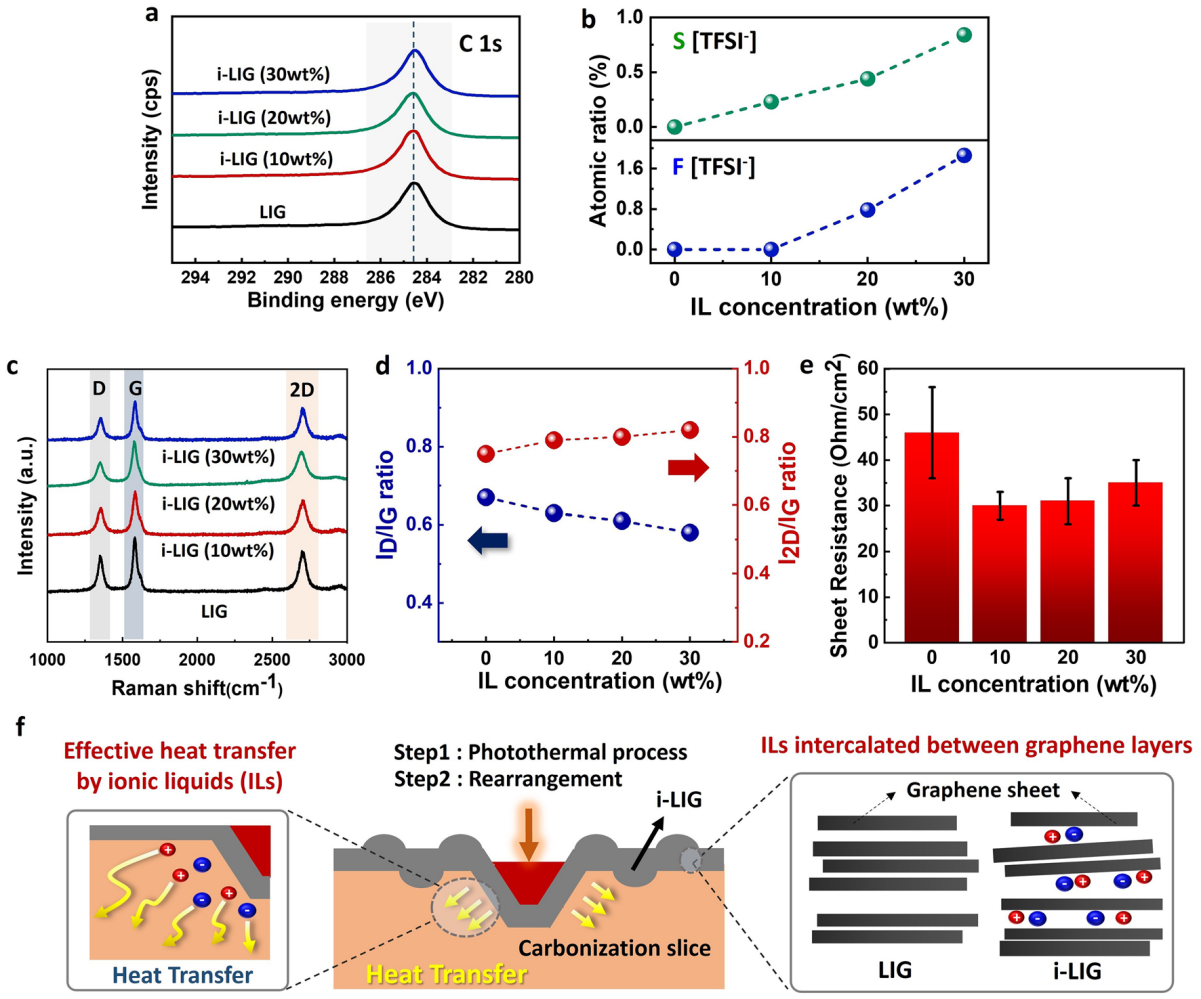
Characterization of i-LIGs with varying concentrations of ILs (10–30 wt%) (**a**) High-resolution XPS C1s spectrum of pristine LIG and i-LIGs with varying concentrations of ILs (10–30 wt%). (**b**) Elemental composition analysis, including elements such as S and F in i-LIGs with varying concentrations of ILs (10–30 wt%). (**c**) Raman spectra of pristine LIG and i-LIGs with varying concentrations of ILs (10–30 wt%). (**d**) Analysis of I_D_/I_G_ and I_2D_/I_G_ ratios with varying concentrations of ILs (10–30 wt%). (**e**) Electrical resistance measurements of i-LIGs with varying concentrations of ILs (10–30 wt%). (**f**) Schematic illustration of the growth mechanism of i-LIGs through the photothermal carbonization and rearrangement process.

We proceeded with the synthesis of i-LIG electrodes on a PI ion-gel, systematically assessing the quality of graphene through structural and electrical analyses (Fig. 3). The synthesis conditions for i-LIG were precisely tailored by fine-tuning the CO_2_ laser power settings, as depicted in Figure S3 for PI neat. Initially, we verified the degree of graphitization in i-LIG through XPS analysis of carbon (C), fluorine (F), and sulfur (S). As illustrated in Fig. 3a, the C1s spectra of both i-LIG and LIG exhibited a distinctive C=C peak at 284.6 eV, devoid of oxidation peaks (C–O, C=O). This indicates the formation of a robust graphene lattice with sp^2^ carbon bonds, unaffected by the presence of the ion gel. Additionally, the i-LIG electrode showed an increasing trend in the atomic ratio of S and F elements induced by the ion gel at higher concentrations (Fig. 3b)^23^. This increase is due to ion intercalation on graphene layers during LIG synthesis, resulting in the formation of graphene with fewer layers and superior electrical characteristics. Moreover, we investigated the crystallinity of i-LIG electrodes at various ion gel concentrations using Raman spectroscopy. The recorded Raman spectra exhibited three predominant peaks: D, G, and 2D peaks at 1350 cm^−1^, 1580 cm^−1^, and 2700 cm^−1^, respectively (Fig. 3c). Typically, the ratios of these peaks (D/G = I_D_/I_G_ and 2D/G = I_2D_/I_G_) signify the degree of defects and the number of graphene layers^24–26^.

Figure 3d shows a reduction in the I_D_/I_G_ ratio for the 30 wt% i-LIG electrode compared to pristine LIG, indicating enhanced crystallinity and reduced defects in i-LIG derived from the PI ion gel. Additionally, the I_2D_/I_G_ ratio exhibits an increasing trend as the ion concentration within the ion gel rises, suggesting the generation of lower graphene layers facilitated by the ion gel's intercalation effect between the graphene layers. Beyond its exceptional structural qualities, the i-LIG electrode demonstrates approximately 30% lower sheet resistance than pristine LIG, emphasizing superior electrical conductivity compared to LIG derived from PI neat (Fig. 3e). The outstanding graphene quality and electrical properties of our i-LIG electrodes are elucidated through the synthesis mechanism of LIG electrodes. Typically, the synthesis of LIG involves a two-step process, comprising a laser photothermal process and rearrangement^27,28^. Similarly, the growth mechanism of i-LIG can be elucidated as a two-step process, as illustrated in Fig. 3f and Figure S4. Upon CO_2_ laser irradiation of the PI ion gel, the surface temperature of the ion gel rapidly rises, exceeding the range of 2500–3000K^29^. This temperature elevation results from localized lattice vibrations induced by laser energy with Gaussian spatial distribution. In this thermal process, chemical bonds like C–O, C=O, and C–N undergo cleavage, initiating surface carbonization. Simultaneously, carbon atoms adopt a honeycomb graphite structure, transitioning from sp^3^ to sp^2^ bonding configurations through the rearrangement process, ultimately forming i-LIG. The IL within the PI ion gel enhances heat transfer during these photothermal and rearrangement processes, efficiently promoting the pyrolysis of chemical bonds. Notably, the low vapor pressure of IL and its intercalation effect between graphene layers contribute to a decrease in graphene layers^30,31^. Essentially, IL significantly enhances the effectiveness of both the photothermal and rearrangement processes, resulting in the creation of higher-quality graphene compared to PI neat.

**Figure 4:**
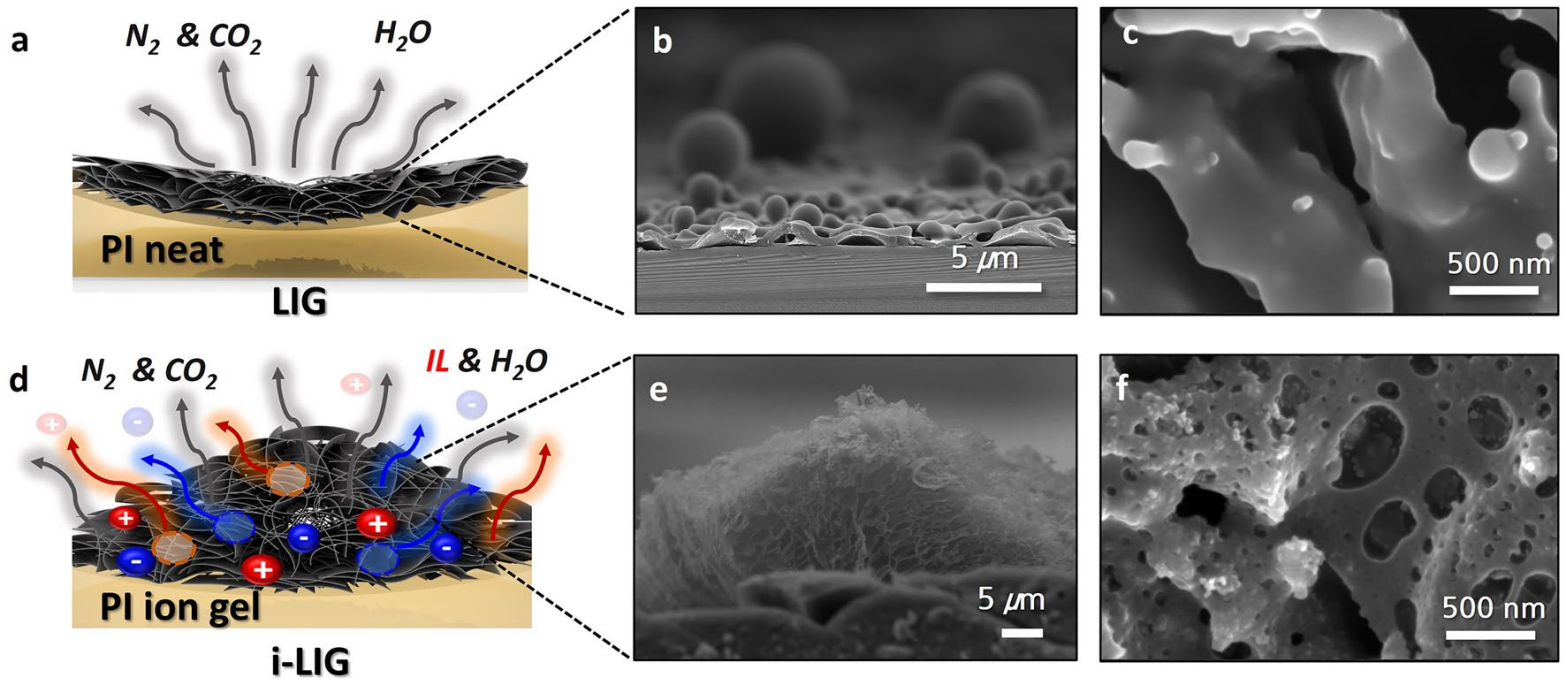
Morphological analysis of i-LIGs. Schematic representations of (**a**) pristine LIG and (**d**) i-LIG with 30 wt% ILs. Cross-sectional FE-SEM images of (**b**) pristine LIG and (**e**) i-LIG with 30 wt% ILs. High magnification surface morphology of (**c**) pristine LIG and (**f**) i-LIG with 30 wt% ILs.

Furthermore, to investigate how IL influences the morphological characteristics of the i-LIG electrode, we conducted a thorough analysis using FE-SEM (Fig. 4, Figure S5). As seen in Fig. 4a and Figure S6, LIG formed from PI neat without ILs exhibits a typical porous structure where chemical bonds undergo cleavage, and substances vaporize into gases during CO_2_ laser irradiation^32^. Consistent with previous studies, the cross-sectional (Fig. 4b) and surface (Fig. 4c) FE-SEM images of LIG reveal a rough surface and porous structure. In contrast, in the case of the PI ion-gel, an expanded porous structure and enlarged three-dimensional electrode morphology are observed with an increasing IL concentration (Fig. 4d, Figure S6). This is attributed to the photothermal process where the IL increases the carbonizable volume, facilitating efficient heat transfer and IL vaporization at specific temperatures, resulting in a larger porous structure in i-LIG compared to LIG. Particularly, in the 30 wt% PI ion gel, IL maximizes heat transfer and ion vaporization, leading to a significantly increased porous structure and expanded electrode morphology (Fig. 4e and f). The expanded electrode structure has the potential to enhance EDL capacitor formation by providing a more extensive interface with the PI ion gel.

**Figure 5:**
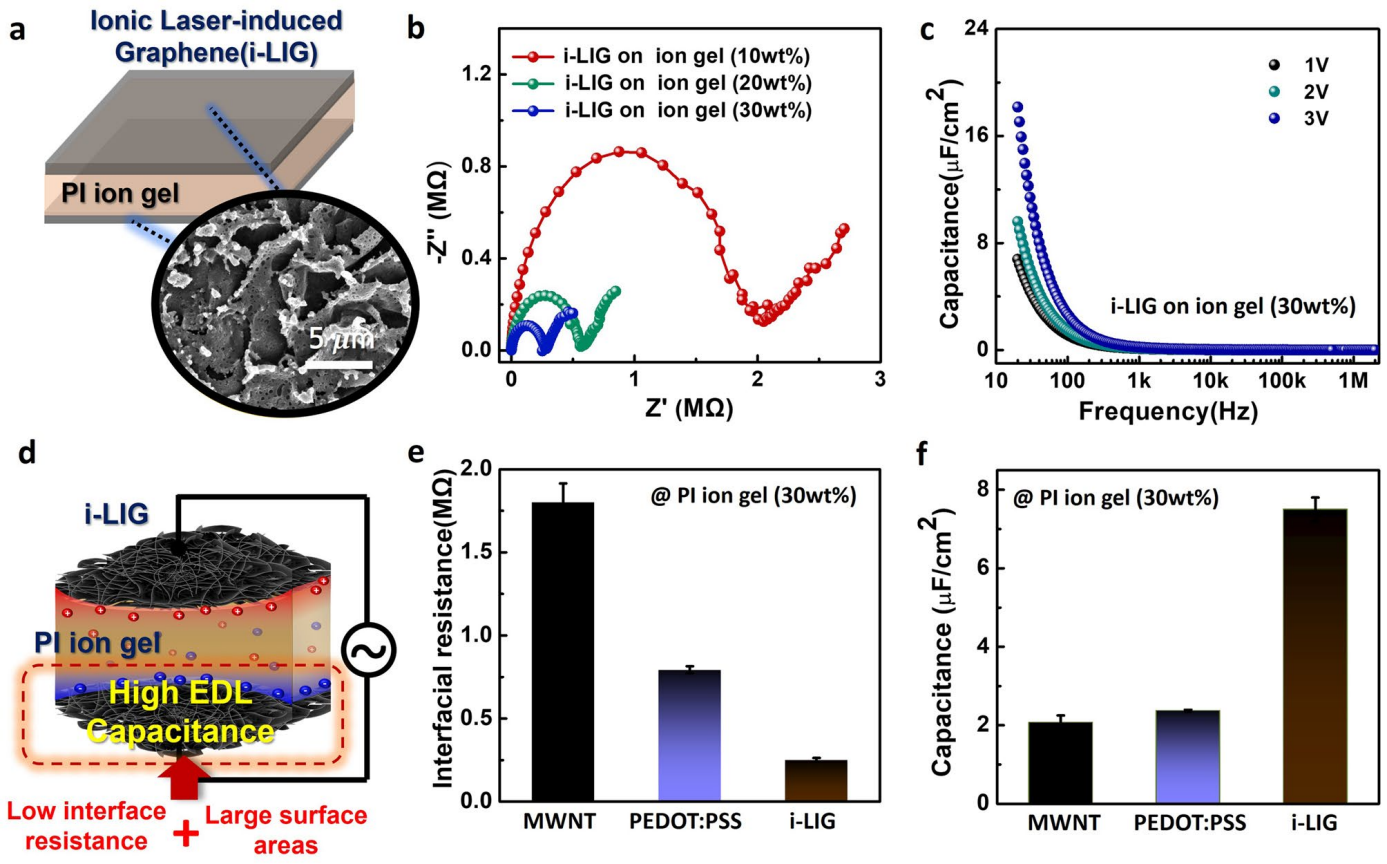
Electrical and electrochemical characteristics of a vertical capacitor with i-LIG and PI ion gel (**a**) Schematic image of a capacitor with i-LIG and PI ion gel. (**b**) Impedance Nyquist plots (imaginary part − Z′′ as a function of real part Z′) of i-LIG on PI ion gel with varied amount of ILs (10–30 wt%). (**c**) Capacitance-frequency of i-LIG on ion gel with 30 wt% ILs. (**d**) An illustration image of the operating characteristics of the vertical capacitor with i-LIG and PI Ion Gel (**e**) Interface resistance of PI ion gel with ILs (30 wt%) depending on electrode. (**f**) Capacitance characteristics of PI ion gel with ILs (30 wt%) depending on electrode. Each error bar represents the standard deviation around the mean.

Finally, we explored interfacial properties between PI ion gels and i-LIG electrodes and their potential as iontronic devices (Fig. 5). Figure 5a depicts the structural schematic of a vertical capacitor designed to confirm the interfacial properties of PI ion gel and i-LIG. This capacitor forms upper and bottom electrodes on the surface of the PI ion gel through CO_2_ laser irradiation. As shown in Fig. 5b, the higher the IL concentration of the vertical capacitor composed of PI ion gels and i-LIGs, the lower the interfacial resistance. Additionally, the capacitance of the vertical capacitor with a 30 wt% IL content increased with decreasing frequency and increasing applied voltage, reaching a high capacitance value of 18 μF/cm^2^ at 3 V voltage (Fig. 5c). The capacitor utilizing i-LIG and PI ion gel not only exhibits exceptional characteristics as individual components of electrodes and ion gel but also highlights the critical role of an effective interface in forming significant EDL capacitance in iontronics. Ultimately, the stable interface between ion gels and i-LIG facilitates high EDL capacitance, driven by low interfacial resistance between electrodes and PI ion gels, coupled with a broad interfacial area facilitating easy ion accumulation (Fig. 5d). To experimentally validate this principle, we selected MWNT and PEDOT:PSS as representative solution process-based electrode materials for comparison. To ensure a consistent comparison of resistance among the i-LIG electrode and the control groups (MWNT and PEDOT:PSS electrodes), we utilized electrodes with different thicknesses: the average thickness of MWNTs was 3 µm, PEDOT:PSS was 800 nm, and i-LIG was 7 µm.

The two electrodes were spray-coated on PI ion gel (30 wt%) to produce the same structure as the vertical capacitor based on i-LIG electrodes, and electrical and electrochemical characteristics were compared and analyzed. The impedance graphs revealed comparable initial electrical resistance values for the three electrodes, including two comparison groups (MWNT: 1.68 kΩ, PEDOT:PSS: 1.58 kΩ, i-LIG: 1.76 kΩ). However, the interfacial resistance on the PI ion gel, derived from the diameter of the semi-circle in the impedance graphs, decreased in the order of MWNT, PEDOT:PSS, i-LIG, with i-LIG exhibiting the lowest interfacial resistance of 0.3 MΩ (Fig. 5e). This indicates that i-LIG forms a stable interface with the PI ion gel compared to the other two electrodes. When examining capacitance values, the order of increase was PEDOT:PSS, MWNT, i-LIG, with i-LIG recording a capacitance of 8 μF/cm^2^, more than four times higher than that of MWNT(Fig. 5f). These results demonstrate that i-LIG exhibits superior EDL capacitance due to low interfacial resistance and a significant interfacial area between the electrode and ion gels. In summary, the exceptional EDL capacitance characteristics of i-LIG electrodes highlight their versatile potential in low-voltage driven high-performance iontronic devices compared to other flexible electrodes.

In conclusion, we have successfully demonstrated the direct bottom-up synthesis of an i-LIG electrode by irradiating a CO_2_ laser onto a PI ion gel, confirming stable interfacial formation capable of establishing an EDL. The designed PI ion gel exhibited robust mechanical properties, maintaining stability regardless of ion concentration. Furthermore, an increase in ion concentration led to a noticeable enhancement in EDL capacitance, attributed to improved ion migration behavior. Additionally, with an increase in the concentration of IL in the PI ion gel, the resulting i-LIG demonstrated high crystallinity and excellent electrical properties, indicating the synthesis of high-quality laser-induced graphene. This can be attributed to the efficient heat transfer characteristics of ILs within the PI ion gel and their role as intercalation materials between graphene layers. In conclusion, we constructed a vertical capacitor using PI ion gel/i-LIG, exhibiting superior ion migration behavior and electrical characteristics. Electrochemical analysis confirmed the EDL formation characteristics. Experimentally, it was demonstrated to have significantly lower interfacial resistance and higher EDL capacitance compared to the comparative groups of MWNT and PEDOT:PSS. These results highlight the revolutionary possibilities of employing the direct bottom-up synthesis method for the i-LIG electrode system using PI ion gel. This not only pioneers a new direction in flexible iontronics research but also holds the promise of making significant contributions to future advancements in the field.

## Methods

### Fabrication of PI ion gels and i-LIGs

To produce PI ion gels, amic acid solution (Poly(pyromellitic dianhydride-co-4,4'-oxydianiline), 12.8 wt%, Sigma-Aldrich) and [EMIM]^+^[TFSI]^-^ (1-Ethyl-3-methylimidazolium bis(trifluoromethylsulfonyl)imide, ≥ 98% (HPLC), Sigma-Aldrich) were utilized. Various weight percentages of [EMIM]^+^[TFSI]^-^, specifically 10, 20, and 30 wt% relative to the amic acid solution, were added and mixed for 1 h using a paste mixer, resulting in a uniform precursor solution. The fabrication of PI ion gels can be achieved through two distinct methods, spin coating and pouring, depending on the desired film morphology. Firstly, the solution was spin-coated onto a substrate (wafer or PI film), and the solvent was gradually removed on a hotplate, ranging from 60 °C to 120 °C at a rate of 10 °C/hour for 24 h, resulting in solid PI ion gels. Secondly, the solution was poured into a petri dish, and the solvent was removed on a hotplate, ranging from 60 °C to 100 °C for 24 h, forming solid PI ion gels. For the formation of ionic laser-induced graphene (i-LIG), direct laser irradiation of the PI ion gels was conducted using a 10.6 mm CO_2_ laser. The conditions for CO_2_ laser irradiation for i-LIG formation were optimized by controlling the power of the CO_2_ laser (Power: 20%).

### Mechanical characterization of PI ion gel

The mechanical properties were obtained by conducting three measurements for each sample using a dynamic mechanical analyzer (DMA850, TA Instruments). In terms of tensile test, all specimens were cut into rectangular shape (5 mm*20 mm). The deformation was determined at a strain rate of 5 mm/min. For these samples, Young’s modulus was determined in the linear region with a low strain of less than 1%.

### Structural and chemical characterizations of PI ion gel and i-LIGs

The crystallinity of the PI ion gels were analyzed by X-ray diffraction (XRD) analysis (Rigaku). Chemical bonding and elemental analysis of the i-LIGs were conducted using X-ray photoelectron spectroscopy (XPS, Thermo scientific, ESCA Probe). XPS spectra were recorded with normal emission geometry using monochromatic Al Kα radiation (hν = 1486.6 eV) in an ultrahigh vacuum system (pressure: ~ 10^−9^ Torr) with a pass energy of 50.0 eV. Structural features of the i-LIGs were characterized by Raman spectroscopy (in Via Raman microscope, Renishaw). The morphological features of the i-LIGs were characterized by FE-SEM (JSM-6700F, JEOL) and scanning electron microscopy (SEM, S-4700, Hitachi).

### Electrical and electrochemical characterizations of PI ion gel and i-LIGs

The sheet resistance of the i-LIGs were measured by 4-point probe measurement system (CMT-SR1000N, Advanced Instrument Technology). Capacitance measurements were conducted using a precision LCR meter (E4980a, Agilent Technologies). The electrical characteristics of the PI ion gels and vertical capacitors with i-LIGs/PI ion gels were evaluated using an LCR meter (E4980a, Agilent Technologies). Electrochemical impedance spectroscopy (EIS) was conducted using an electrochemical analyzer (PGSTAT302N, Metrohm Autolab) over a frequency range of 0.1 Hz to 1 MHz with a 10 mV AC signal at room temperature.

### Supplementary Information

Supplementary Information.

## Data Availability

All data are included in this article and its Supplementary Information files. All data are available from the corresponding authors upon reasonable request.
