# Supplementary material for: Highly conductive, conformable ionic laser-induced graphene electrodes for flexible iontronic devices
Source: Sci Rep. 2024 Feb 26;14:4599. doi: 10.1038/s41598-024-55082-w (PMC10897153; doi:10.1038/s41598-024-55082-w)
Supplement: Supplementary file 1 — Supplementary Information. [file 41598_2024_55082_MOESM1_ESM.pdf]

Supplementary Information

Highly Conductive, Conformable Ionic Laser-induced Graphene Electrodes for Flexible Iontronic Devices

So Young Kim[1,5+], Ji Hong Kim[1,+], Kyeong Nam Kim[2,+], Hayoung Oh[1],
Sung Myung[3,*], and Do Hwan Kim[1,4,5*]

[1]Department of Chemical Engineering, Hanyang University, Seoul, 04763, Republic of Korea
[2]Division of Energy Technology, DGIST, Daegu, 42988, Republic of Korea
[3]Thin Film Material Research Center, Korea Research Institute of Chemical Technology (KRICT), Daejeon, 34114, Republic of Korea
[4]Institute of Nano Science and Technology, Hanyang University, Seoul 04763, Republic of Korea
[5]Clean-Energy Research Institute, Hanyang University, Seoul 04763, Republic of Korea

*Corresponding authors: Do Hwan Kim (dhkim76@hanyang.ac.kr); Sung Myung (msung@krict.ac.kr).
[+]These authors contributed equally to this work

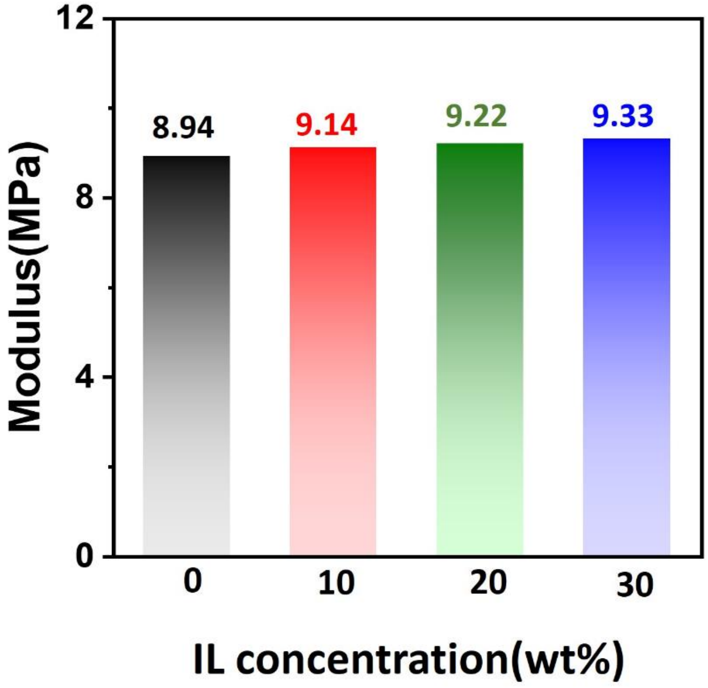

Supplementary Figure S1. Mechanical properties of PI ion gel with different ILs contents (0-30wt%).

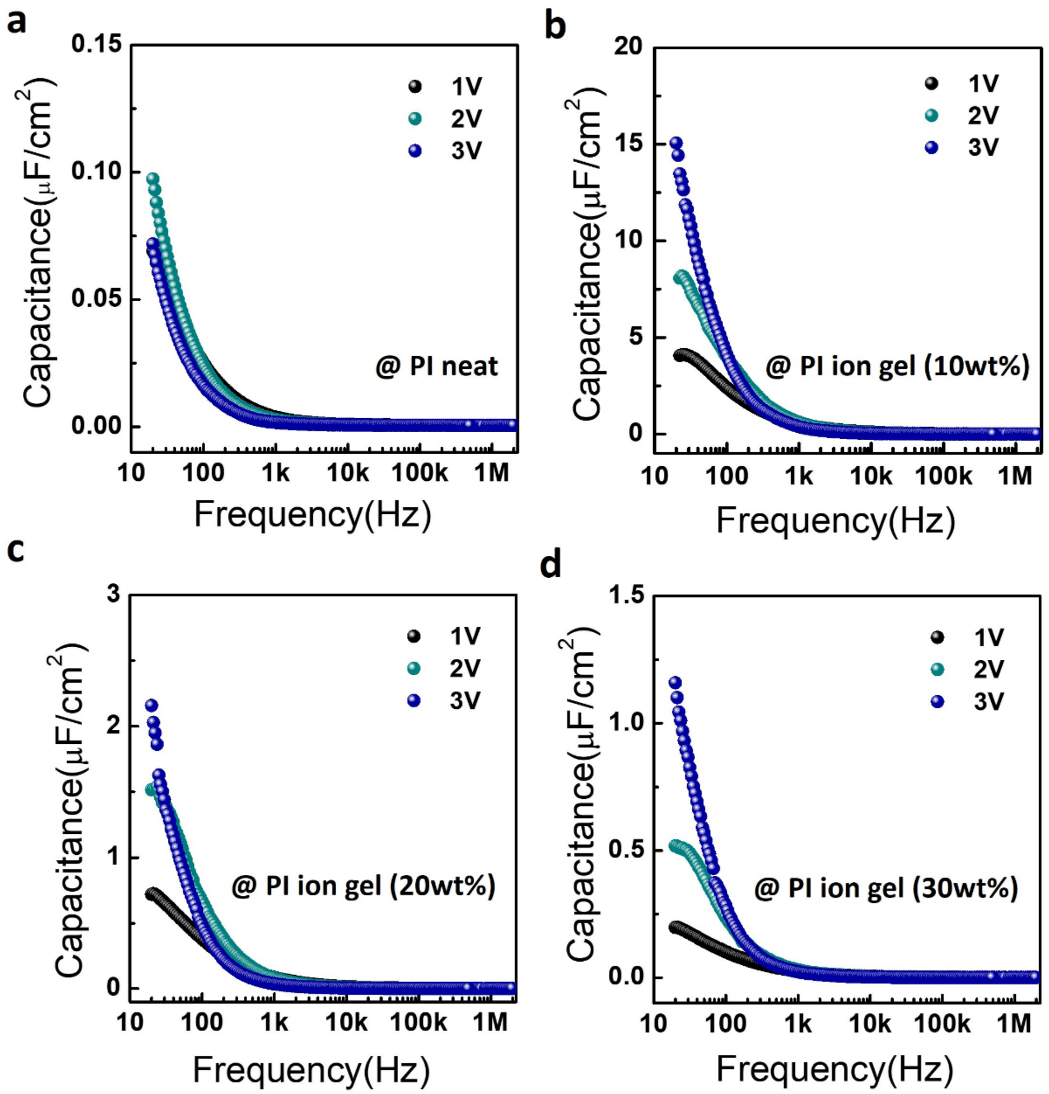

Supplementary Figure S2. Capacitance-frequency of PI ion with varying concentrations of ILs (0-30 wt%). (a) PI neat film (b) PI ion gel with 10wt% of ILs (c) PI ion gel with 20wt% of ILs (d) PI ion gel with 30wt% of ILs .

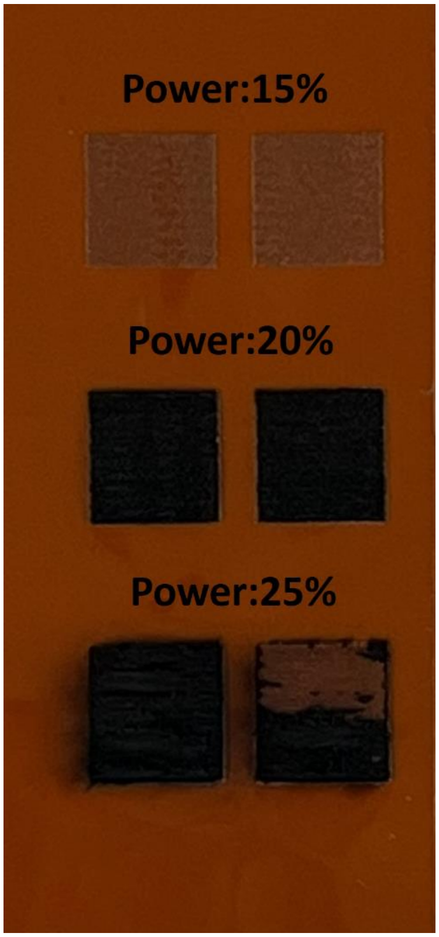

Supplementary Figure S3. Photograph of i-LIG with varying CO_2 laser power (15%, 20%, 25%).

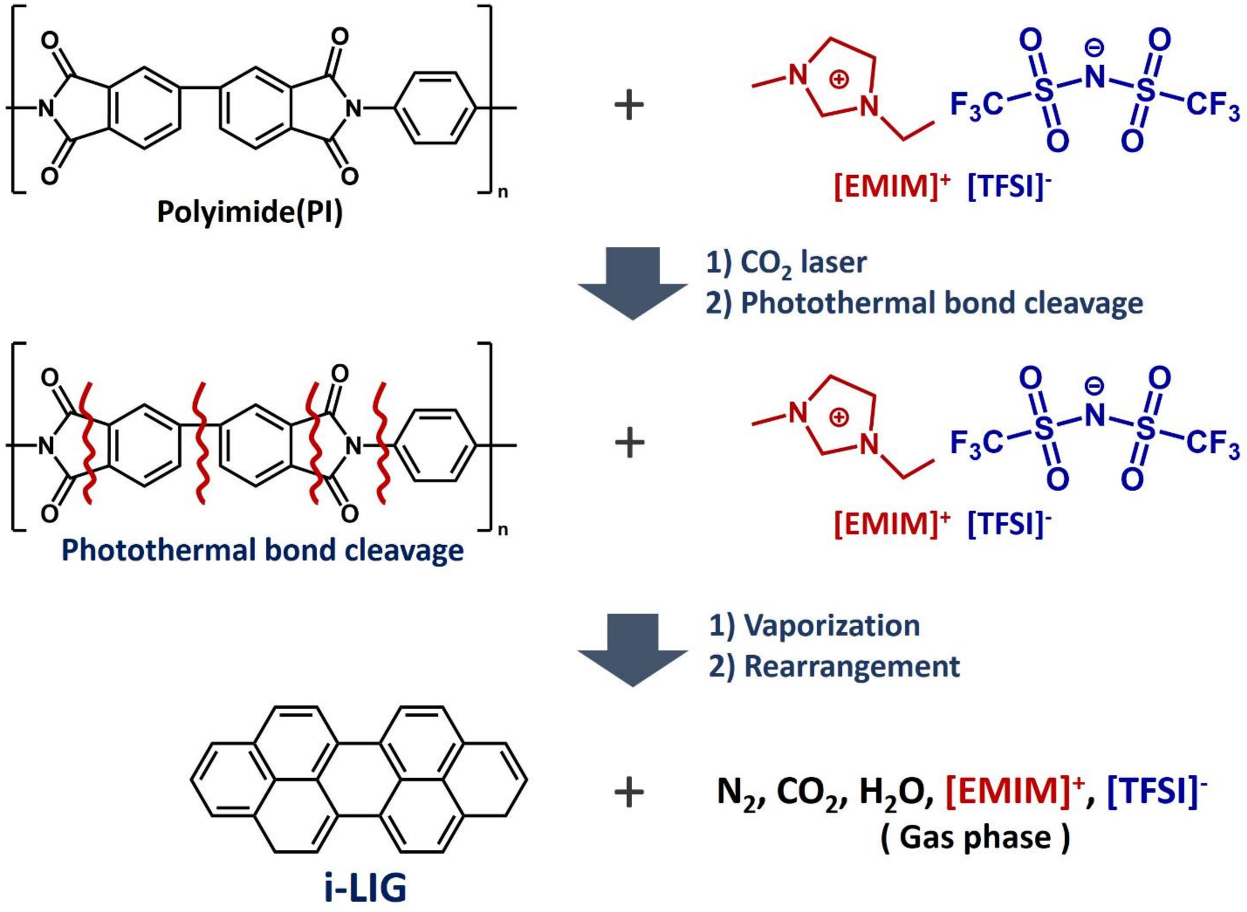

Supplementary Figure S4. Mechanism schematic of conversion LIG from PI ion gel.

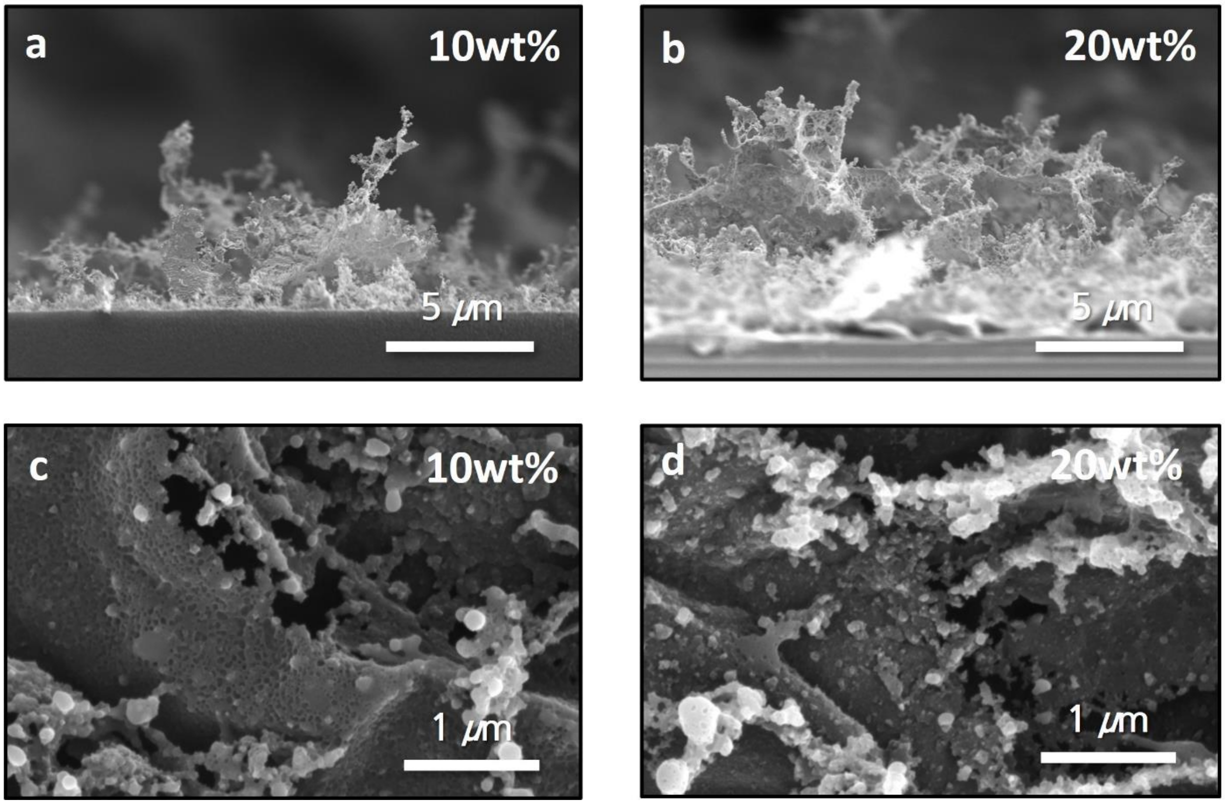

Supplementary Figure S5. Morphological analysis of i-LIGs. Schematic representations Cross-sectional SEM images of (a) i-LIG with 10 wt% of ILs and (b) i-LIG with 20 wt% of ILs. Top view SEM images of (c)) i-LIG with 10 wt% of ILs and (d)) i-LIG with 20 wt% of ILs.

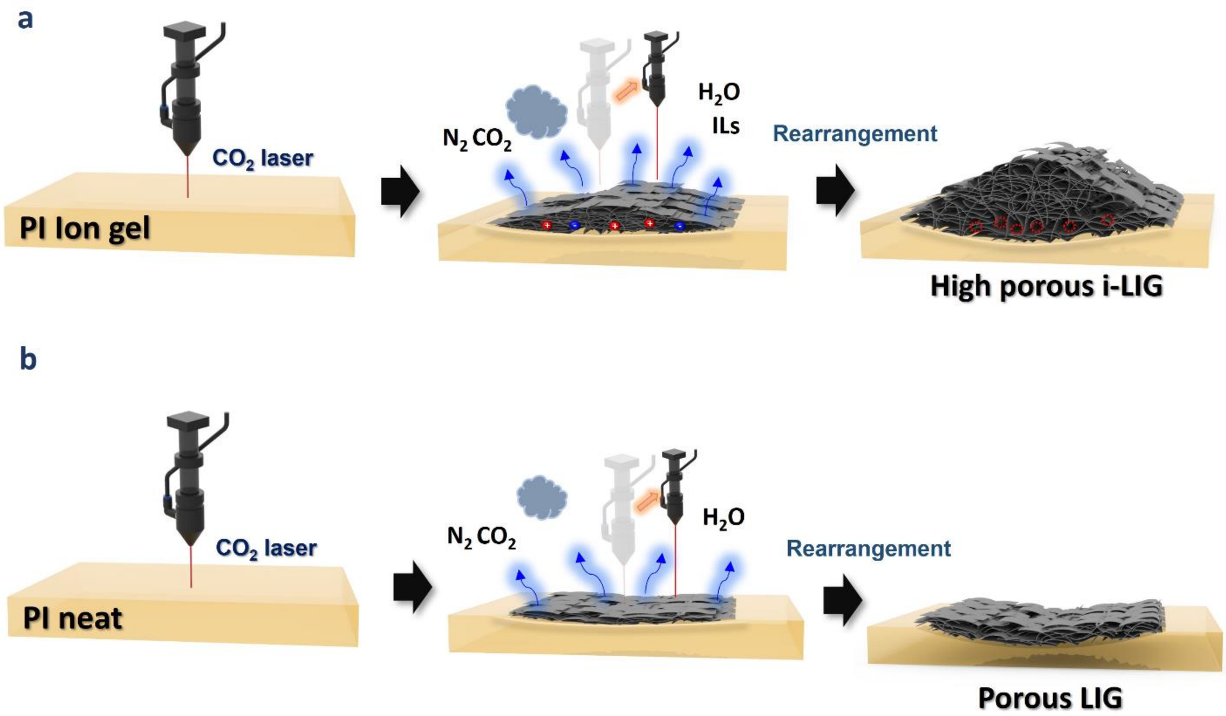

Supplementary Figure S6. Mechanism schematic of producing LIG mechanism from (a) PI ion gel and (b) PI neat
